# Autism-associated gene Dlgap2 mutant mice demonstrate exacerbated aggressive behaviors and orbitofrontal cortex deficits

**DOI:** 10.1186/2040-2392-5-32

**Published:** 2014-05-01

**Authors:** Li-Feng Jiang-Xie, Hsiao-Mei Liao, Chia-Hsiang Chen, Yuh-Tarng Chen, Shih-Yin Ho, Dai-Hua Lu, Li-Jen Lee, Horng-Huei Liou, Wen-Mei Fu, Susan Shur-Fen Gau

**Affiliations:** 1 Department of Psychiatry, National Taiwan University Hospital and College of Medicine, No. 7, Chung-Shan South Road, Taipei 10002, Taiwan; 2 Graduate Institute of Brain and Mind Sciences, National Taiwan University, Taipei, Taiwan; 3 Department of Psychiatry, Chang Gung Memorial Hospital-Linkou, Taoyuan, Taiwan; 4 Department and Graduate Institute of Biomedical Sciences, Chang Gung University, Taoyuan, Taiwan; 5 Department of Pharmacology, School of Medicine, College of Medicine, National Taiwan University, 11F No.1 Sec. 1, Ren-Ai Road, Taipei 10051, Taiwan; 6 Department of Anatomy and Cell Biology, College of Medicine, National Taiwan University, Taipei, Taiwan

**Keywords:** *Dlgap2*, aggressive behavior, orbitofrontal cortex, autism, synapse, mouse model

## Abstract

**Background:**

As elegant structures designed for neural communication, synapses are the building bricks of our mental functions. Recently, many studies have pointed out that synaptic protein-associated mutations may lead to dysfunctions of social cognition. *Dlgap2*, which encodes one of the main components of scaffold proteins in postsynaptic density (PSD), has been addressed as a candidate gene in autism spectrum disorders. To elucidate the disturbance of synaptic balance arising from *Dlgap2* loss-of-function *in vivo*, we thus generated *Dlgap2*^*−/−*^mice to investigate their phenotypes of synaptic function and social behaviors.

**Methods:**

The creation of *Dlgap2*^*−/−*^mice was facilitated by the recombineering-based method, Cre-loxP system and serial backcross. Reversal learning in a water T-maze was used to determine repetitive behaviors. The three-chamber approach task, resident–intruder test and tube task were performed to characterize the social behaviors of mutant mice. Cortical synaptosomal fraction, Golgi-Cox staining, whole-cell patch electrophysiology and transmission electron microscopy were all applied to investigate the function and structure of synapses in the orbitofrontal cortex (OFC) of *Dlgap2*^*−/−*^mice.

**Results:**

*Dlgap2*^*−/−*^mice displayed exacerbated aggressive behaviors in the resident–intruder task, and elevated social dominance in the tube test. In addition, *Dlgap2*^*−/−*^mice exhibited a clear reduction of receptors and scaffold proteins in cortical synapses. *Dlgap2*^*−/−*^mice also demonstrated lower spine density, decreased peak amplitude of miniature excitatory postsynaptic current and ultra-structural deficits of PSD in the OFC.

**Conclusions:**

Our findings clearly demonstrate that *Dlgap2* plays a vital role in social behaviors and proper synaptic functions of the OFC. Moreover, these results may provide valuable insights into the neuropathology of autism.

## Background

Social behavior is the evolutionary foundation of our complex society and culture but its genetic basis is still an enigma [1]. One of the greatest scientific challenges in modern neuroscience is to unveil the mystery of the social genetic basis of social behaviors. As social behaviors are widely observed in the animal kingdom, and their patterns share many similarities across species, it is desirable to elucidate the evolutionary conserved genetic bases of social behaviors [2]. In humans, impaired social reciprocity is one of the core symptoms of autism [3] and aggressive behavior, which is a manifestation of social dysfunction frequently observed in individuals with autism [4]. Genetic epidemiological studies have already demonstrated that autism has a definitely strong genetic basis [5]. Pinpointing the genes that are critical for social behaviors will not only improve our scientific knowledge but also shed light on targets for clinical intervention for patients with autism. Recently, several large-scale human genetic studies have clearly demonstrated that mutations of synaptic proteins can lead to social dysfunctions [6-9]. Synapses, across which neurons transmit, exchange and process information, are the building bricks of our mental function. A specialized group of synaptic macromolecules, the postsynaptic scaffolding proteins, which play a role as the master organizers of macromolecular assembly within the postsynaptic density (PSD), is pivotal to proper synaptic functions [10,11]. DLGAP2 (also known as SAPAP2 or GKAP2), as one of the main components of postsynaptic scaffolding proteins, directly interacts with DLG4 (also known as PSD-95) and SHANKs to form the DLG4-DLGAPs-SHANKs complex, which plays critical roles in synaptic morphogenesis and functions [12-14]. Human genetic studies point out that mutations of synaptic scaffold proteins may contribute to the etiology of psychiatric and neurodevelopmental disorders [15,16]. Further, *DLG4*[17], *SHANKs*[18-20] and *DLGAP2*[8,21,22] have been listed as possible candidate genes for autism. According to the findings of Pinto *et al.*, *DLGAP2* was encompassed in rare *de novo* copy number variations, which were not found in the control group [8]. In our previous study of copy number variation, we also identified a patient carrying a *de novo* 8p23.2-pter microdeletion, which encompasses *DLGAP2*[23]. Several genetically manipulated mouse models have been used to demonstrate successfully that disruptions of *Dlg4*[17] and *Shanks*[24-27] can lead to abnormalities in social behaviors. However, the role of *Dlgap2* still remains elusive, which intensifies our eagerness to unveil whether *Dlgap2* regulates social behaviors or synaptic functions.

Here, we report that *Dlgap2*^*−/−*^mice displayed elevated aggressive behaviors, a socially dysfunctional behavior, in the resident–intruder task and enhanced social dominance in the tube test. Using biochemical, electrophysiological and ultra-structural studies, we found that *Dlgap2*^*−/−*^mice exhibit pronounced synaptic deficits in the orbitofrontal cortex (OFC), a brain region that plays a critical role in behavioral inhibition and regulating aggressive drives. Our results may provide valuable insights into the neural mechanisms of social behaviors and autism.

## Methods

### Mice

**Figure 1:**
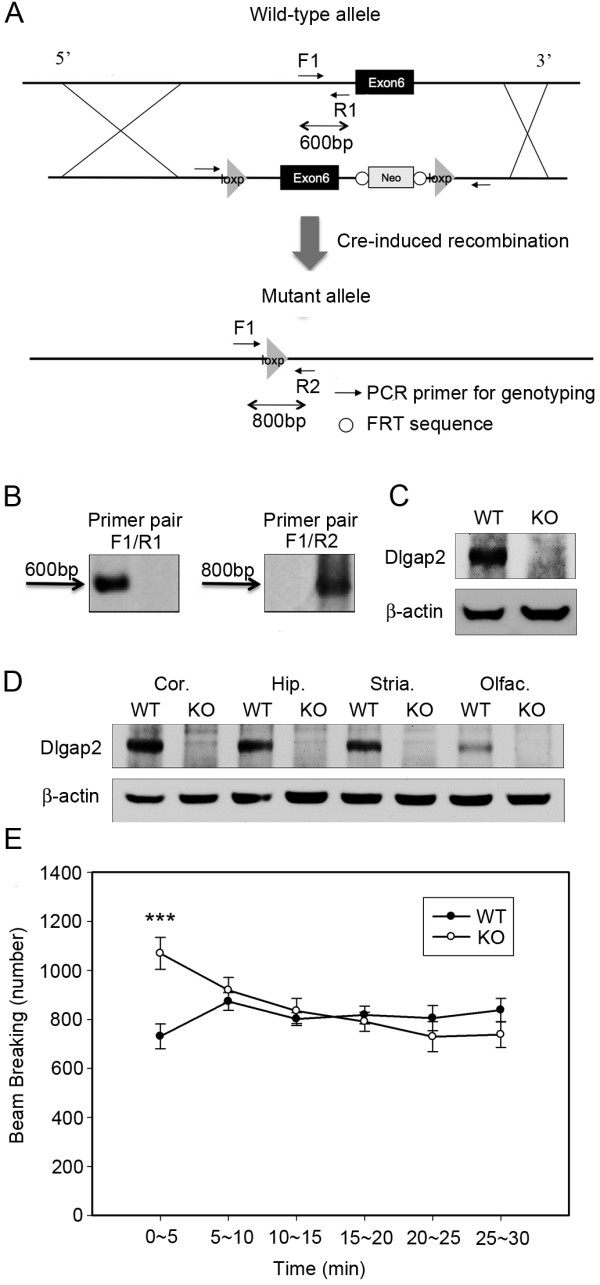
**Generation of *Dlgap2***^***−/− ***^**(KO) mice. (A,B)** Deletion of exon 6 in the *Dlgap2* gene. **(C)** The whole brain lysate of *Dlgap2*^*−/−*^mice lacked DLGAP2 protein products. **(D)** DLGAP2 was detected in the cortex (cor.), hippocampus (hip.), striatum (stria.) and olfactory bulb (olfac.) of *Dlgap2*^*+/+*^ (WT) mice, but not in *Dlgap2*^*−/−*^mice. **(E)***Dlgap2*^*−/−*^mice displayed novelty-induced hyperactivity in the open-field test. Data are presented as mean ± standard error of the mean. *n* =11 for WT and 10 for KO mice. ***P < 0.001 from a two-tailed *t*-test. bp, base pair; cor., cortex; F1, forward primer 1; hip., hippocampus; KO, knockout; olfac., olfactory bulb; PCR, polymerase chain reaction; R1, reverse primer 1; R2, reverse primer 2; stria., striatum; WT, wild type; FRT, flippase recognition target.

We used the method of targeting vector construction to generate *Dlgap2* knockout (KO) mice as previously described by Liu, Jenkins, and Copeland [28]. In summary, exon 6 of *Dlgap2* (Gene ID: 244310) in embryonic stem (ES) cells from the 129S1/Sv mouse strain was replaced by a construct containing *Dlgap2* exon 6 interposed between two loxP sites and a NEO cassette via spontaneous homologous recombination. Exon 6 of *Dlgap2* was knocked out by Cre-induced homologous recombination (Figure 1A). The ES cell clones containing the mutant allele were rechecked by Southern blot DNA fragment analysis to ensure the correctness of gene targeting. The targeted ES cells were microinjected into the blastocysts from the C57BL/6 mouse strain and the injected blastocysts were transferred into ICR foster mothers to produce chimeric mice. F1 founders were produced by mating male chimeric mice with wild-type (WT) C57BL/6 females. The mice were backcrossed for more than eight generations to make the congenic strain in a 99.9% C57BL/6 genetic background. Combinations of forward primer F1 (GCCACATTCATAACATAGCTAC), reverse primer 1 (R1) (ACCTC TGCTACATACCCACTC) and reverse primer 2 (R2) (ACACATGGGATGCTGTACGC) were used to determine the genotype of each mouse. The amplicon of mutant allele was 800 bp and the amplicon of the WT allele was 600 bp (Figure 1B). The congenic strain *Dlgap2* KO mice and its WT littermates were used for analysis in this study.

### Locomotion test

Adult male mice were allowed to explore freely for 30 minutes in a square (16 × 16 inches) open-field arena (San Diego Instruments, San Diego, CA USA). Locomotion activity was monitored using a 16 × 16 photo-beam sensor. The numbers of beam-breaking events were computed as an index of locomotion activity.

### Three-chamber test

Social approach and social novelty tests were performed as previously described with minor modifications [29-31]. A Plexiglas cage (40 × 60 × 22 cm) was divided into three equal regions (40 × 20 × 22 cm). At the beginning of the test, a mouse was allowed to habituate in the central chamber for 10 minutes. We then removed the doorway between the chambers to let the mouse acclimate to the environment for another 10 minutes. For the sociability test, we first confined the test mouse in the central chamber again, and then put one small Plexiglas cylinder to constrain a target mouse in one side chamber and an empty Plexiglas cylinder in the third chamber. The Plexiglas cylinders have numerous holes to facilitate social communications. To enhance the behavioral measure sensitivity, we defined the small square space (20 × 20 cm) containing the Plexiglas cylinder with a mouse as the social region, and the space with the empty Plexiglas cylinder as the object region. After the removal of the doorway, the time spent in each region by the test mouse was automatically recorded for 5 minutes. When the test was over, the mice were allowed to explore the chambers freely for another 5 minutes. For the social novelty test, a novel mouse was then put in the previously empty Plexiglas cylinder. The time spent in each region was automatically recorded for 5 minutes again. Group-housed adult male mice (8 to 9 weeks of age) were used in these experiments.

### Resident–intruder task

Adult male mice (8 weeks of age) from both genotypes were single-housed in their home cages for 3 weeks. On the test day, an adult male intruder with similar body weight was placed into the cage. For the initial 5 minutes of social contact, aggressive behaviors were scored by a rater blinded to the genotypes. The aggressive behavioral indexes, include biting, wrestling, tail-rattling, aggressive grooming and chasing, were as previously described [32].

### Tube test

The tube test was performed as previously described with minor modifications [33,34]. We designed transparent Plexiglas tubes with a length of 30 cm and inside diameter of 3.2 cm. This narrow space is just sufficient for a mouse to walk through without being able to reverse its direction. Mice were trained to walk through the tube before testing. On the test day, mice from both genotypes were released at opposite ends of the Plexiglas tube, and we made sure that they met at the middle of the tube. The mouse that retreated first from the tube was defined as the loser and the other mouse, which stayed in the tube, was the winner.

### Reversal learning in water T-maze

The reversal learning in water T-maze test [35] was modified from the method described by Gusariglia and Chadman [36]. The water T-maze consists of a white Plexiglas T-maze, which is placed in a circular pool with a diameter of 100 cm. The pool was filled with water to a depth of about 33.5 cm at 22 ± 0.5°C. The Plexiglas T-maze consists of a start arm (30 × 10 cm) and two goal arms (30 × 10 cm) with walls (20 cm high). A platform (Plexiglas, 5 × 5 cm) was placed at the end of one goal arm and submerged 1 cm below the surface of the water. The apparatus was set in a room without visual cues. Data were collected manually by a single observer.

Mice were placed in the water T-maze without the platform to acclimate them to the task environment for 60 s as pre-training. In the habit acquisition session, a platform was located at the end of only one goal arm. Each mouse was placed in the starting arm and allowed up to 60 s to find the submerged platform. When a mouse failed to find the platform in 60 s, it was picked up by the experimenter and placed onto the platform for 10 s. Mice underwent ten trials per day. On each trial, an error was recorded when the mouse entered the arm without the platform or entered the arm with the platform and left that arm. Only when the mouse entered the arm and climbed onto the platform directly was this recorded as a correct choice. When a mouse had been trained to perform eight out of ten trials correctly for four consecutive days, it was subjected to training for reversal learning. In the reversal learning session, the platform was switched to the opposite arm of the T-maze. The training procedure was the same as the acquisition session. The entry into the arm where the platform was originally located was recorded as an error. The total numbers of errors per day were measured throughout the 4-day reversal learning session.

### Synaptosomal fraction preparation and Western blot

Crude synaptosomal fractions of mouse cerebral cortex were prepared as previously described in Schmeisser *et al.*’s work [25]. In brief, a cortex was quickly dissected out and homogenized in HEPES-buffered sucrose (320 mM sucrose, 5 mM 4-(2-hydroxyethyl)-1-piperazineethanesulfonic acid (HEPES), pH 7.4) containing proteinase inhibitor cocktail. The homogenized cortices were then centrifuged at 1,000 *g* for 10 minutes at 4˚C to remove cell debris and nuclei. Then, the supernatant was spun at 12,000 *g* for 20 minutes to yield the soluble and the crude synaptosomal fractions. Equal amounts of protein per lane from both genotypes were separated by SDS-PAGE. Primary antibodies used in immunoblot experiments included NR1 (Abcam, Cambridge, MA USA), NR2A (Abcam), NR2B (Abcam), GluR1 (Millipore, Billerica, MA USA), GluR2 (Abcam), Shank3 (Abcam), PSD95 (Abcam), Homer-1b/c (Santa Cruz, Dallas, Texas USA), αCaMKII (Santa Cruz), βCaMKII (Abcam), β-actin (Cell Signaling, Danvers, MA USA) and Dlgap2 (GeneTex, Hsinchu Taiwan). After incubation with the primary antibodies, peroxidase-conjugated secondary antibodies were applied to the blots and incubated for 1 hour at room temperature. The blots were visualized with a chemiluminescence reagent using Biospectrum Imaging System (UVP, Upland CA). The quantity of each protein was normalized for the quantity of the corresponding β-actin detected in the sample.

### Golgi-Cox stain for spine density

Dendritic spines were counted in layer II/III pyramidal cells in the OFC using the Golgi-Cox stain. In brief, brain tissues were taken and placed in the impregnation solution (solutions A and B in FD Rapid GolgiStain kit, FD NeuroTechnologies, Ellicott City, MD, USA) for 17 days. After several washes, brain tissues were cut into coronal sections with a vibratome to a thickness of 150 μm. Brain sections were collected and reacted with the mixture of developer and fixer in FD Rapid GolgiStain kit (solutions C and D, 1:1) for 2 minutes. Dendritic spines were examined with a 100× objective oil immersion lens and captured with the Stereoinvestigator system (Microbrightfield, Williston, VT USA). For quantitative analysis of spine density, the spines were counted along dendritic segments chosen from secondary and tertiary dendrites using the Image J software. The experimenter was blind to genotype and selected one to three segments for each cell.

### Electrophysiology

The animal-use protocol was approved by the Animal Ethics Committee of the National Taiwan University, Taiwan. Briefly, animals were sacrificed by decapitation and the fresh brains were quickly removed into chilled (0 to 4°C) cutting solution containing: 0.5 mMCaCl_2_, 110 mM choline chloride, 25 mM glucose, 2 mM KCl, 7 mM MgSO_4_, 26 mM NaHCO_3_, 1.25 mM NaH_2_PO_4_, 11.6 mM sodium ascorbate and 3.1 mM sodium pyruvate. Coronal brain slices (250 to 300 μm in thickness) containing the OFC were cut by a microslicer (Dosaka DTK-1000, Ted Pella, Altadena, CA USA) then transferred to a holding chamber with artificial cerebrospinal fluid consisting of: 2 mM CaCl_2_, 10 mM glucose, 3 mM KCl, 1 mM MgCl_2_, 125 mM NaCl, 26 mM NaHCO_3_ and 1.25 mM NaH_2_PO_4_. Slices were then maintained at room temperature for at least 1 hour before recording and bubbled with 95% O_2_ and 5% CO_2_. A brain slice was transferred to the recording chamber and held in position using a stainless steel grid with a nylon mesh and submerged in a solution at 1 to 2 ml/min. Whole-cell recordings were made from layer II/III OFC pyramidal neurons using a patch-clamp amplifier (Axopatch 200B, Molecular Devices, Sunnyvale, CA USA). Neurons were visualized under infrared-differential interference contrast lens and digital camera by an upright microscope (BX51WI, Olympus, Tokyo, Japan). Patch electrodes (3 to 8 MΩ) were pulled from borosilicate glass capillaries by a micropipette puller (P97, Sutter Instrument, Novato, CA USA) and filled with a solution containing: 140 mM K-Glu, 5 mM KCl, 10 mM HEPES, 0.2 mM EGTA (ethylene glycol tetra-acetic acid), 2 mM MgCl_2_, 4 mM MgATP, 0.3 mM Na_2_GTP (guanosine-5'-triphosphate, disodium salt) and 10 mM Na_2_-phosphocreatine, pH 7.2 (with KOH). Data acquisition was performed using a digitizer and pClamp 10 software (DigiData 1440A, Molecular Devices). A presynaptic stimulation was carried out with a glass pipette filled with 3 M NaCl solution placed in layer I of the prelimbic region of the recording site using a stimulator (S-48, Grass-Telefactor, Warwick, RI USA) and an isolation unit (ISO-Flex, AMPI, Jerusalem, Israel). To calculate the paired-pulse ratio (PPR), two stimuli pulses were delivered at 25-, 50- or 100-ms intervals and the PPR was measured by dividing the second excitatory postsynaptic current (EPSC) by the first. Data were excluded from the analysis if the input resistance varied >20% throughout the experiment. To isolate the α-amino-3-hydroxy-5-methyl-4-isoxazolepropionic acid receptor-miniature excitatory postsynaptic currents (AMPAR-mEPSCs), a cell was clamped at -70 mV in the presence of tetrodotoxin (1 μM), picrotoxin (100 μM) and APV (amino phosphonovalerate, 50 μM) and analyzed using MiniAnalysis Program (Synaptosoft, Decatur, GA USA). Signals were filtered at 2 Hz and digitized at 10 Hz.

### Electron microscopy

Adult male mouse brains were transcardially perfused with fixative (2% paraformaldehyde and 2% glutaraldehyde in 0.1 M phosphate buffer). Subsequently, the brains were dissected out and post-fixed in the same fixative as described above overnight at 4°C. We then used a vibratome to section the mouse frontal cortex into 300 μm thicknesses, and isolated the region of interest, the OFC. These samples were further post-fixed in 1% osmium tetroxide for 1 hour at 25°C. The samples were then dehydrated and embedded in epoxy or spurr resin. Ultrathin sections of 70 nm were cut using an ultramicrotome. After lead citrate staining, these sections were examined by electron microscopy. Images were acquired at 40,000× magnification, and PSD thickness and length were measured with ImageJ.

## Results

### Generation of Dlgap2^−/−^mice

We generated *Dlgap2*^*−/−*^mice (Figure 1A,B) by deleting exon 6 from the gene, the largest coding exon of the *Dlgap2* gene. The deletion generated a frame-shift mutation that resulted in complete functional loss of DLGAP2 protein, due to inadequate translation of the C-terminal. With Western blotting analysis, we further confirmed that mutant mice lacked DLGPA2 protein products (Figure 1C). It is known that *Dlgap2* is highly expressed in the cortex, striatum, hippocampus and olfactory bulbs [14,37], as a result, we could only detect its protein products in these brain regions of WT mice but not those of the mutants (Figure 1D). *Dlgap2*^*−/−*^mice had similar body shapes and weights as their WT littermates (Additional file 1: Figure S1). To assess their basic locomotion ability, we used the open-field test (Figure 1E) and found that *Dlgap2*^*−/−*^mice demonstrated intact locomotion ability since their performances were quite comparable to the WT mice. However, *Dlgap2*^*−/−*^mice displayed novelty-induced hyperactivity (Figure 1E) in the first 5 minutes of the test specifically, which was likely due to impulsivity [38].

### Exacerbated aggressive behaviors of Dlgap2^−/−^mice

**Figure 2:**
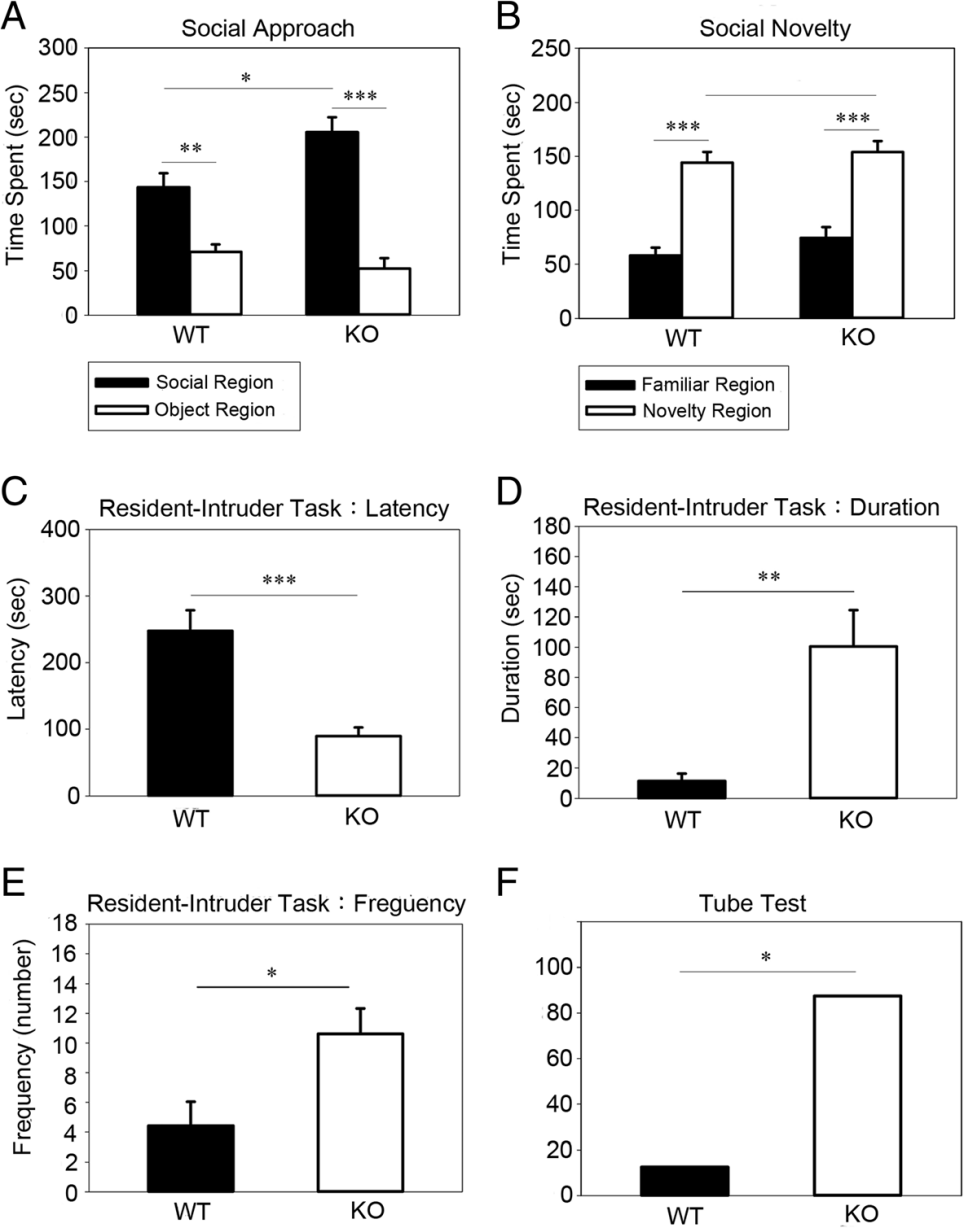
**Abnormal social behaviors in *Dlgap2***^***−/− ***^**mice. (A-B)** Social approach and novelty tests, *n* = 8 per genotype. *Dlgap2*^*−/−*^mice had an intact social preference, spending a longer time spent in the social region, and intact social novelty performance. **(C-E)** Resident–intruder task, *n* =7 *Dlgap2*^*+/+*^ (WT) and 8 *Dlgap2*^*−/−*^ (KO) mice. *Dlgap2*^*−/−*^mice had a shorter attack latency, and exhibited more prolonged and increased frequency of aggressive behaviors. **(F)***Dlgap2*^*−/−*^mice had a higher probability of winning in the tube test, *n* = 8 pairs of mice. Data are presented as mean ± standard error of the mean. *P < 0.05, **P < 0.01 and ***P < 0.001 from two-tailed *t*-tests for **(A)**, **(B)** and **(E)**, Mann–Whitney U test for **(C)** and **(D)**, and chi-squared test for **(F)**. KO, knockout; WT, wild type.

Because *Dlgap2* has been linked to human social dysfunction, we performed a modified version of the three-chamber social approach test to investigate the social behaviors of the mutant mice [29,30]. Initially, a test mouse was left to explore and interact with a strange mouse held in a plastic cage or with an empty plastic cage without any mice inside. Both genotypes manifested clear preference for social stimulus, indicating normal sociability (Figure 2A). However, on examining the data more carefully, we can see that *Dlgap2*^*−/−*^mice seemed to display enhanced social approach behavior relative to the WT littermates since they spent significantly more time in the social chamber (Figure 2A). In the subsequent experimental trial, a new stimulus mouse was introduced into the previously empty cage to see if the test mice would interact with the familiar or novel mouse. Since mice have a natural tendency to explore novelty, they prefer to investigate the novel mouse if they are able to distinguish and recognize it. Our results demonstrated that both genotypes showed an intact and similar social recognition ability in the social novelty test (Figure 2B).

Although *Dlgap2*^*−/−*^mice seemed to display normal sociability, the motivations behind the approaching behaviors could not be easily discerned by the three-chamber task. For example, social affiliation might be a positive motivation for mice to approach others; however, aggression may be another factor in motivating the mice to approach. We thus used the resident–intruder task to evaluate the aggressiveness of the *Dlgap2*^*−/−*^mice. Briefly, mice were single-housed in their home cage for 3 weeks to establish their territory. On the test day, intruders were put in to induce aggressive behaviors in the resident mice. As the results show, *Dlgap2*^*−/−*^mice displayed an obviously shorter attack latency (Figure 2C). Additionally, the duration and frequency of aggressive behaviors also dramatically increased compared to the WT mice (Figure 2D,E).

To provide further evidence of elevated aggression, we used the tube dominance test. Mice from each genotype were released into the opposite ends of a narrow tube. The more dominant mouse aggressively forces the opponent mouse out of the tube. The mouse to step out of the tube first was identified as the loser. Our results clearly manifested that the *Dlgap2*^*−/−*^mice had a much higher probability of winning, indicating elevated aggression (Figure 2F).

### Reversal learning deficit of Dlgap2^−/−^mice

**Figure 3:**
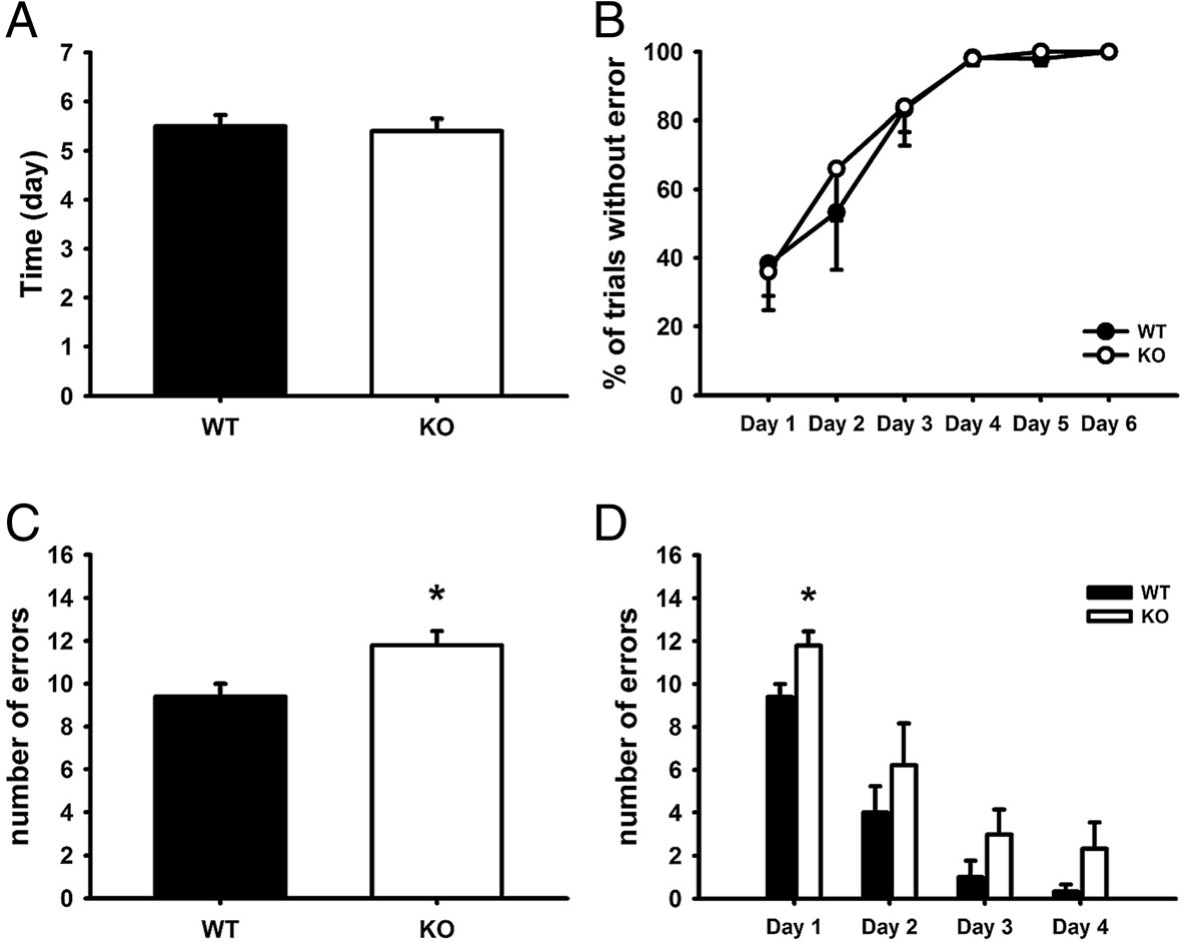
**Reversal learning deficit in *Dlgap2***^***−/− ***^**mice.** The water T-maze test was performed as described in Methods. **(A)** Days needed to reach criteria for reversal learning in the habit acquisition session. **(B)** Percentage of correct trials without errors throughout the 6-day habit acquisition session. Note that there was no difference between WT and *Dlgap2*^*−/−*^KO mice in the acquisition phase. **(C)** Total number of errors made on Day 1 of reversal learning session. Note that more errors were made by *Dlgap2* KO mice on the first day of reversal learning. **(D)** Total numbers of errors made during the 4-day reversal learning session. Data are presented as mean ± standard error. *n* = 5 for each group. * *P* < 0.05 compared with WT. KO, knockout; WT, wild type.

We used performance in the water T-maze to determine the repetitive behaviors of *Dlgap2*^*−/−*^mice. As shown in Figure 3A, there was no significant difference in the number of days to reach the 80% right position criterion between WT and *Dlgap2*^*−/−*^mice (5.5 and 5.4 days, respectively). All mice reached the criterion on Day 6 and were moved into training session of reversal learning test (Figure 3B). In the learning session of reversal learning test, we found that *Dlgap2*^*−/−*^mice made a significantly greater number of errors on the first day of learning session in comparison with WT mice (*P* < 0.05) (Figure 3C). Figure 3D shows the number of errors on each day throughout the 4-day reversal learning session. During reversal learning, both strains improved across days.

### Disruptions of synapse in orbitofrontal cortex of *Dlgap2*^*−/−*^mice

**Figure 4:**
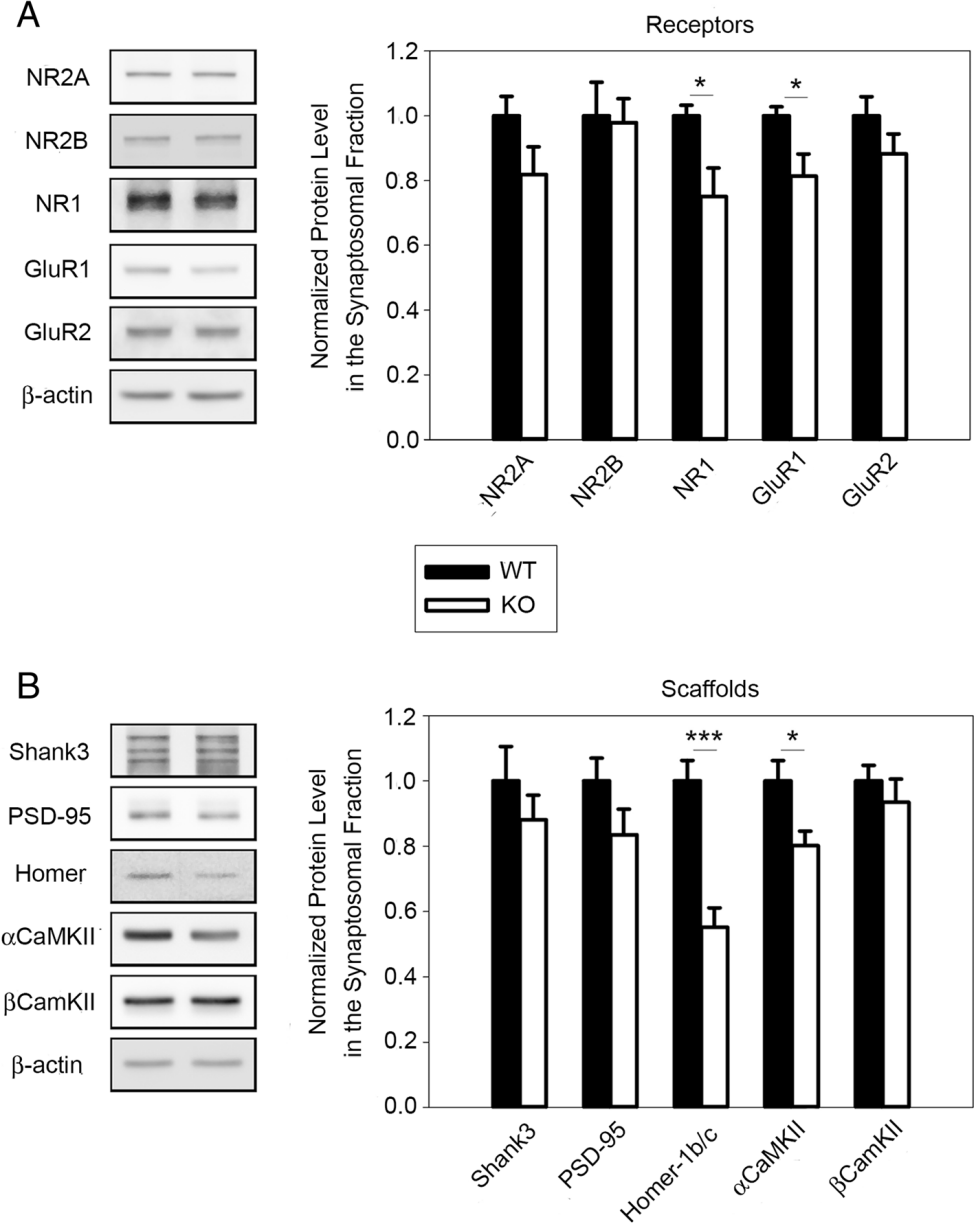
**Downregulation of synaptic scaffold proteins and receptors in the cortex of *Dlgap2***^***−/− ***^**mice. (A)** Quantities of synaptic receptor proteins NR1 and GluR1 were decreased in cortical synaptosomal fractions from *Dlgap2*^*−/−*^mice. **(B)** Quantities of synaptic scaffold proteins Homer-1b/c and αCaMKII were reduced in cortical synaptosomal fractions from *Dlgap2*^*−/−*^mice. Data are presented as mean ± standard error of the mean. *n* =6 for each genotype. *P < 0.05 and ***P < 0.001 from a two-tailed *t*-test. KO, knockout; WT, wild type.

The cerebral cortex plays a critical role in social cognition and inhibition of aggressive drives [39,40]. Given that DLGAP2 is a synaptic protein highly expressed in the cortex, and the integrity of Dlg4-DLGAP-SHANKs may play critical roles in synaptic structure and functions, we isolated crude synaptosomal fractions from the cerebral cortex and analyzed the protein composition. There was a clear downregulation of synaptic receptors GluR1 and NR1 in mutants, which may indicate that synaptic transmission is impaired (Figure 4A). Moreover, the levels of synaptic scaffold proteins Homer-1b/c and αCaMKII were also lower in the cortical synaptosomal fractions, which may imply a disruption to synaptic structures (Figure 4B). Based on these results, we reasoned that there are severe synaptopathies within the cerebral cortex of *Dlgap2*^*−/−*^mice.

**Figure 5:**
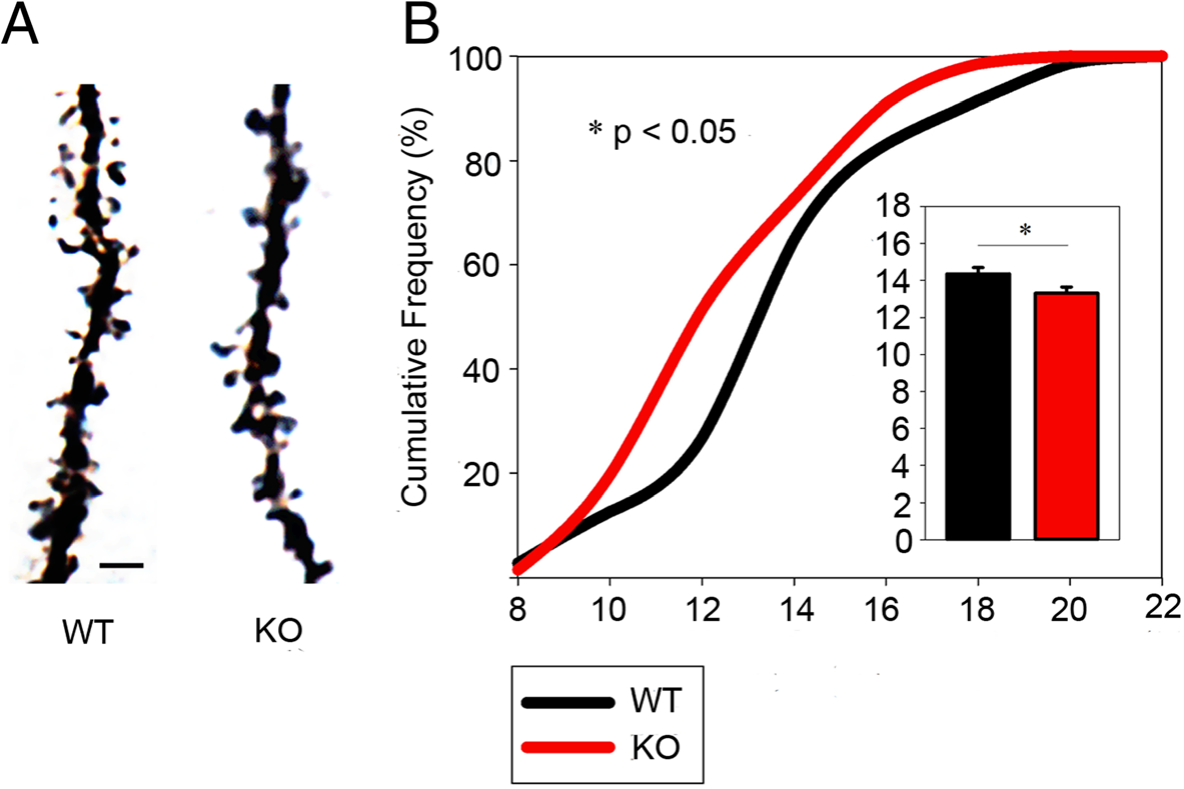
**Reduction of spine density in orbitofrontal cortex of *Dlgap2***^***−/− ***^**mice. (A)** Representative pictures of spine density from *Dlgap2*^*+/+*^ (WT) and *Dlgap2*^*−/−*^ (KO) mice. **(B)** Spine density was lower in *Dlgap2*^*−/−*^ (KO) mice. Scale bar: 2.5 μm. Data are presented as mean ± standard error of the mean. *n* = 64 to 70 segments of dendrites from four *Dlgap2*^*+/+*^ (WT) and four *Dlgap2*^*−/−*^ (KO) mice. *P < 0.05 from a two-tailed *t*-test. KO, knockout; WT, wild type.

To further provide a link between cortical synaptopathies and elevated aggressive behaviors, we used the Golgi stain to analyze the spine density of pyramidal neurons in OFC (Figure 5A), which is known to play a crucial role in inhibition of aggressive drives [40]. In human clinical studies and animal studies on primates and rodents, dysfunction of the OFC has been show to cause exacerbated aggression [40]. Furthermore, since DLGAP2 is one of the main protein components of dendritic spines [11], it may regulate synaptogenesis in the OFC. As our experimental results showed, *Dlgap2*^*−/−*^mice demonstrated a clear reduction of spine density (Figure 5B), indicating the pyramidal neurons received less excitable inputs in the OFC.

**Figure 6:**
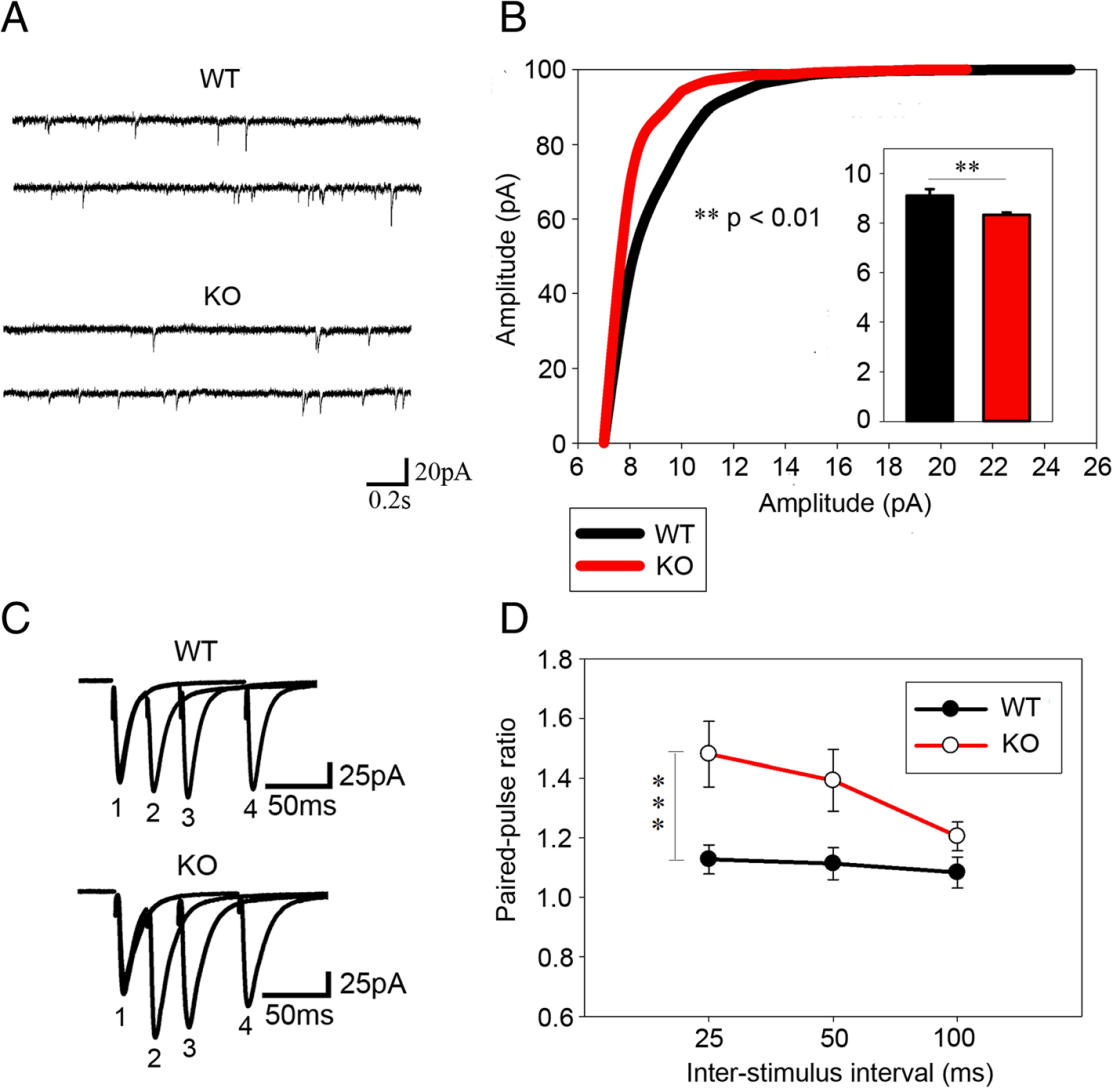
**Deficits of synaptic transmission in orbitofrontal cortex of *Dlgap2***^***−/− ***^**mice. (A)** Example traces of mEPSCs from *Dlgap2*^*+/+*^ (WT) and *Dlgap2*^*−/−*^ (KO) mice. **(B)** Decreased amplitude of mEPSCs in the OFC of *Dlgap2*^*−/−*^mice. *n* = 30 to 35 neurons from five *Dlgap2*^*+/+*^ (WT) and five *Dlgap2*^*−/−*^ (KO) mice. Data are presented as a cumulative frequency curve of mEPSCs and bar graph. **(C)** Representative traces of the paired-pulse ratio from *Dlgap2*^*+/+*^ (WT) and *Dlgap2*^*−/−*^ (KO) mice. The first pulse is indicated by ‘1’. A paired-pulse at 25 ms interval is indicated by ‘2’, a paired-pulse at 50 ms interval by ‘3’ and a paired-pulse at 100 ms interval by ‘4’. **(D)** Paired-pulse ratio, which was enhanced for *Dlgap2*^*−/−*^ (KO) mice. *n* = 7 to 11 neurons per inter-stimulus interval from four *Dlgap2*^*+/+*^ (WT) and three *Dlgap2*^*−/−*^ (KO) mice. *P < 0.05 and ***P < 0.001 from two-tailed *t*-test for **(B)** and two-way ANOVA for **(D)**. KO, knockout; WT, wild type.

Aside from synaptic structure abnormality, electrophysiology experiments were performed to analyze the synaptic functions of *Dlgap2*^*−/−*^mice. We recorded AMPAR-mEPSCs in acute slices of the OFC. Clearly, our results with a lower peak mEPSC amplitude for *Dlgap2*^*−/−*^mice (Figure 6A,B) indicate that the postsynaptic responses of available synapses was weaker functionally. In addition, there was a trend towards a reduction in the frequency of mEPSCs, although this was not statistically significant (Additional file 1: Figure S2). We wondered whether this phenomenon was due to a presynaptic deficit. We thus measured the paired-pulse ratio (PPR). Surprisingly, we found that there was an obvious enhancement of PPR in mutants (Figure 6C,D). Because the PPR of the synaptic response is inversely correlated with presynaptic release probability, our results indicate the release probability of presynaptic vesicles was downregulated. Since DLGAP2 is a scaffold protein located in the PSD, this presynaptic deficit was a surprise. We reason that it was probably mediated by trans-synaptic adhesion molecules [41] or retrograde synaptic signaling [42].

**Figure 7:**
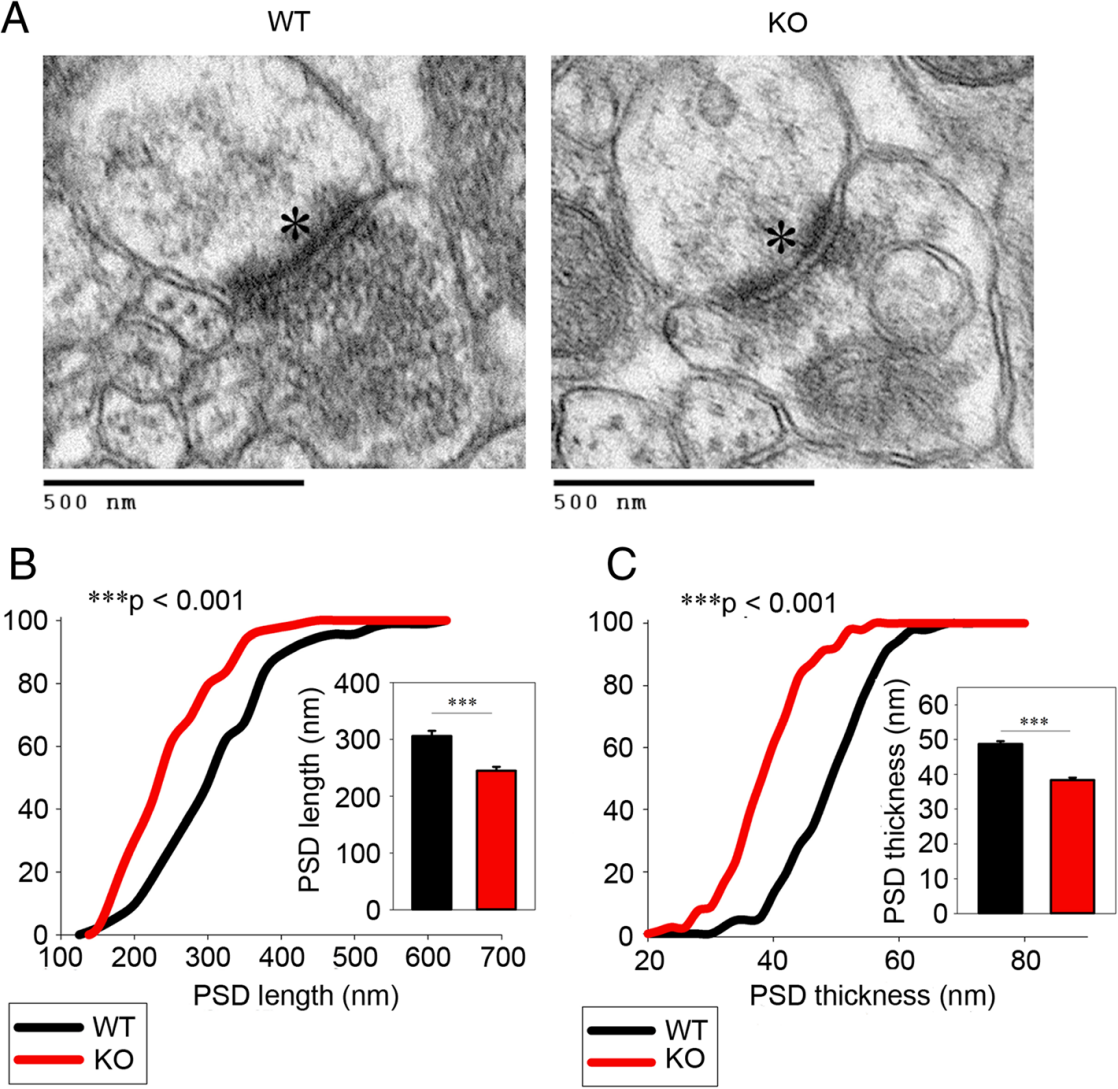
**Ultra-structural deficits in postsynaptic density (PSD) of OFC in *Dlgap2***^***−/− ***^**mice. (A)** Representative electron micrographs showing the PSD (asterisks). **(B)** PSD length, showing its downregulation in *Dlgap2*^*−/−*^mice. **(C)** PSD thickness, showing a decrease for *Dlgap2*^*−/−*^mice. Data in **(B)** and **(C)** are presented as cumulative frequency curves and bar graphs. *n* = 92 to 93 PSDs from three *Dlgap2*^*+/+*^ (WT) and *Dlgap2*^*−/−*^ (KO) mice. ***P < 0.001 from a two-tailed *t*-test. KO, knockout; PSD, postsynaptic density; WT, wild type.

Furthermore, we used transmission electron microscopy to investigate the ultra-structure of synapses in the OFC. Since the morphological integrity of the PSD, which contains vital receptors, scaffolds and signaling molecules, is highly relevant to proper synaptic function, we measured PSD thickness and length (Figure 7). Strikingly, our results demonstrated a clear reduction in the length of the PSD in the OFC (Figure 7B). Additionally, the thickness of the PSD also had dramatic downregulation (Figure 7C). These results indicate the obvious deficits of postsynaptic ultra-structures in *Dlgap2*^*−/−*^mice.

## Discussion

Previous studies have already demonstrated that SHANKs and DLG4, which directly interact with DLGAPs, are involved in regulating murine social behaviors [17,24-26]. The *Dlgap* gene family, encoding isoforms of the SAPAPs, includes *Dlgap1*, *2*, *3* and *4*. It has been demonstrated that the DLGAP family is involved in the pathophysiology of various psychiatric disorders, including Tourette’s syndrome, obsessive–compulsive disorder (OCD), autism spectrum disorders (ASDs) and schizophrenia [8,43-45]. Here we demonstrate that DLGAP2 also plays a vital role in social behaviors. Interestingly, we found that DLGAP2 regulates social behaviors in a totally different way from SHANKs. *SHANK* mutant mice [24-26] have a social withdrawal phenotype; however, *Dlgap2*^*−/−*^mice do not demonstrate social withdrawal but have exacerbated aggressive behaviors. The differential underlying mechanism is worth future investigation. In a broader perspective, these postsynaptic scaffold macromolecules also have direct or indirect molecular interactions with neurexin-neuroligin proteins [41], which contain several autistic candidate genes [46,47]. The neurexin-neuroligin-DLG4-DLGAPs-SHANKs complex thus seems to be a conserved evolutionary mechanism in regulating social behaviors and cognition.

In our study, we nicely prove that *Dlgap2* is vital for normal synaptic structure and functions of the OFC. The OFC has long been implicated in the self-regulation of social-emotional behaviors, which are disrupted in patients with autism [39,48]. In addition, animal studies, in both rodents and primates, indicate that a lesion of the OFC may cause exacerbated aggressive behaviors [40]. Our results may provide the very first link between autistic candidate genes and OFC dysfunction in a murine model. OFC dysfunction has also been implicated in OCD [49] and attention-deficit hyperactivity disorder (ADHD) [50], both of which are psychiatric disorders highly comorbid with ASD. *Dlgap3*^*−/−*^mice have been shown to display obviously OCD-like behaviors [51] and this disease-related phenotype can be rescued by optogenetic stimulation of the OFC-striatal pathway [52]. In addition, a recent human clinical report showed a strong association between *Dlgap2* SNPs and OFC integrity [53]. It would be interesting to analyze OCD-like behaviors systematically in *Dlgap2*^*−/−*^mice in the future.

Repetitive behavior, one of essential features of ASD [54], was also evaluated in this study. In the reversal learning session, *Dlgap2*^*−/−*^mice made a greater number of errors on the first day, indicating an inability to suppress the position habit learned in the previous session. Both WT and *Dlgap2*^*−/−*^mice were equally amenable to position habit learning in the water T-maze assay; therefore, any difference in reversal learning was not caused by overall learning deficits.

Our experimental results provide evidence to support pronounced postsynaptic deficits in the OFC of *Dlgap2*^*−/−*^mice. First, the levels of several postsynaptic proteins were reduced in cortical synaptosomal fractions from mutants (Figure 4). Second, the peak amplitude for AMPAR-mEPSCs was lower in acute slices of the OFC (Figure 5B). Third, the PSD was shorter and thinner in the OFC of the mutants. All of these experiments demonstrated clear-cut postsynaptic deficits in the OFC. On the other hand, we surprisingly discovered that the mutants had obvious presynaptic deficits since there was a clear enhancement of the PPR (Figure 6D), indicating the release probability of presynaptic vesicles was downregulated. Since DLGAP2 is located in the PSD, a charming project will be to elucidate the molecular mechanism of this presynaptic deficit. So far, we postulate it may be mediated by trans-synaptic adhesion molecules [41] or the retrograde synaptic signaling mechanism [42]. It is worthy of mention that *Dlgap3* mutant mice also have an enhanced PPR in striatal medium spiny neurons, which is mediated by retrograde endocannabinoid signaling [42].

## Conclusions

**Figure 8:**
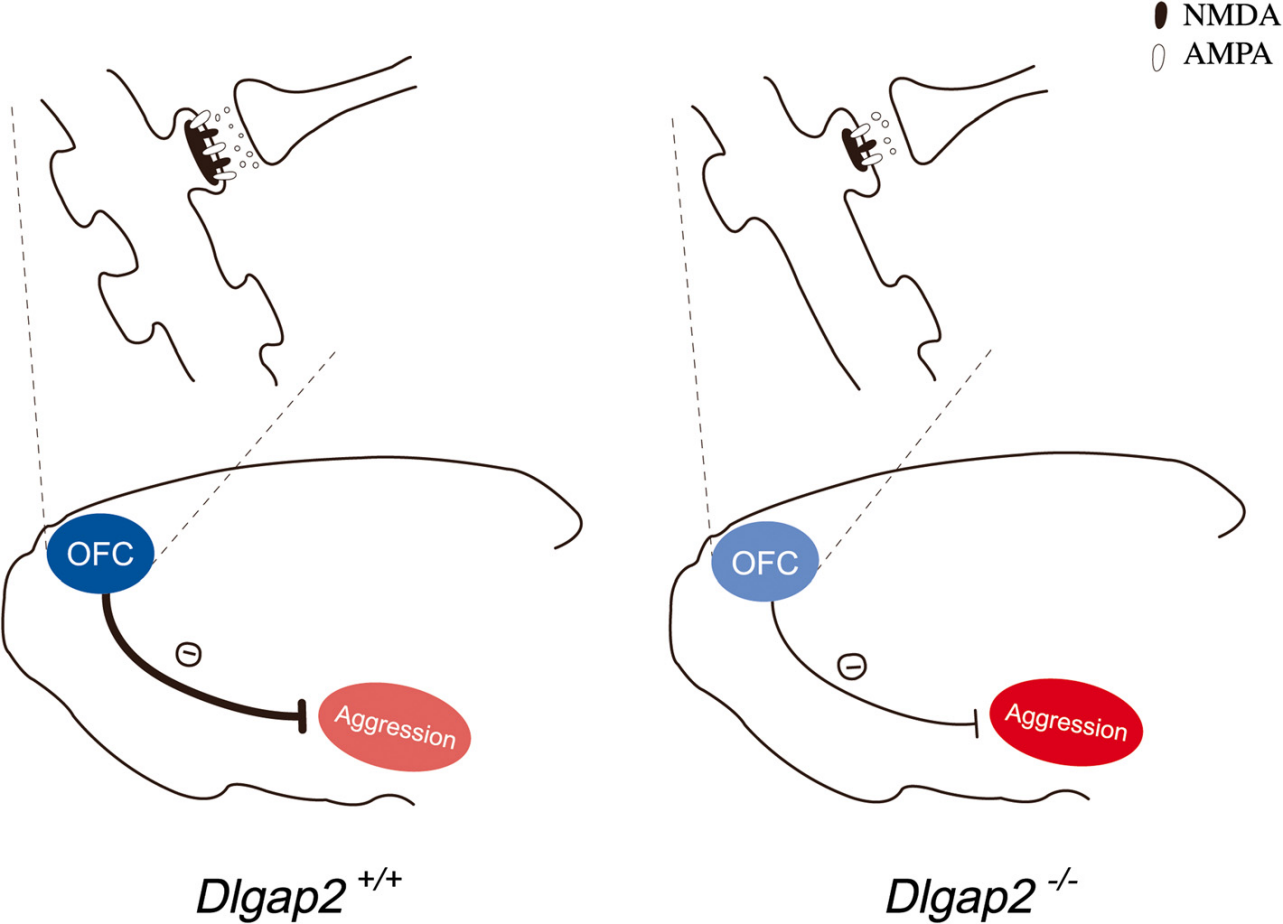
**Working model integrating our experimental results. ***Dlgap2*^*−/−*^mice have shorter and thinner PSDs, fewer receptors allocating on the scaffolds, reduced presynaptic release probability and decreased spine density in the OFC. Due to the obvious disruptions in the OFC, *Dlgap2*^*−/−*^mice display exacerbated aggressive behaviors. AMPA, α-amino-3-hydroxy-5-methyl-4-isoxazolepropionic acid; OFC, orbitofrontal cortex. NMDA, *N*-Methyl-D-aspartic acid.

In conclusion, our findings clearly suggest that DLGAP2 plays a crucial role in maintaining the proper functions of the OFC, a brain region that is implicated in aggressive behaviors. We found that *Dlgap2*^*−/−*^mice have a shorter and thinner PSD in the OFC relative to WT mice. There were clear reductions in the levels of postsynaptic scaffold proteins Homer-1b/c and αCaMKII within the PSD. Synaptic receptors NR1 and GluR1, which are allocated on the scaffolds, also displayed clear downregulation. Because fewer receptors were available on postsynaptic spines, the amplitude of mEPSCs for *Dlgap2*^*−/−*^mice was reasonably reduced. In addition, the release probability of presynaptic vesicles was downregulated, presumably due to trans-synaptic signaling mechanisms. Moreover, the synaptic density for *Dlgap2*^*−/−*^mice was also lower, indicating that there are less excitable inputs into the neurons in the OFC. Our hypothesis is presented in Figure 8. Further impulsivity and aggressive behaviors can be further investigated in *Dlgap2*^*−/−*^mice in the future.

## Abbreviations

ADHD: attention-deficit hyperactivity disorder; AMPAR-mEPSCs: α-amino-3-hydroxy-5-methyl-4-isoxazolepropionic acid receptor-miniature excitatory postsynaptic currents; ASD: autism spectrum disorder; EPSC: excitatory postsynaptic current; ES: embryonic stem; F1: forward primer 1; KO: knockout; mEPSC: miniature EPSC; OCD: obsessive–compulsive disorder; OFC: orbitofrontal cortex; PPR: paired-pulse ratio; PSD: postsynaptic density; R1: reverse primer 1; R2: reverse primer 2; SNP: single nucleotide polymorphism; WT: wild type.

## Competing interests

The authors declare that they have no competing interests.

## Authors’ contributions

SSG initiated and guided this research. SSG, HML and CHC created and verified the mutant mouse. LFJX, YTC and WMF designed the experiments in this paper. LFJX, YTC, DHL and SYH executed the experiments and analyzed the data. LFJX, YTC, DHL, SYH, HHL, LJL, WMF and SSG interpreted the results. LFJX prepared the first draft. HML, CHC, SYH, YTC, WMF and SSG revised the manuscript. HML, WMF and SSG revised the manuscript according to reviewers’ comments. SSG prepared the final files for submission. All authors approved the final version.

## Supplementary Material

Additional file 1: Figure S1 Body weight and shape of *Dlgap2*^*+/+*^ (WT) and *Dlgap2*^*-/-*^ (KO) mice. **Figure S2.** The mEPSC frequency of Dlgap2 *Dlgap2*^*+/+*^ (WT) and *Dlgap2*^*-/-*^ (KO) mice. Click here for file
