# Supplementary material for: Autism-associated gene Dlgap2 mutant mice demonstrate exacerbated aggressive behaviors and orbitofrontal cortex deficits
Source: Mol Autism. 2014 May 1;5:32. doi: 10.1186/2040-2392-5-32 (PMC4113140; doi:10.1186/2040-2392-5-32)
Supplement: Additional file 1: Figure S1 — Body weight and shape of Dlgap2 +/+ (WT) and Dlgap2 -/- (KO) mice. Figure S2. The mEPSC frequency of Dlgap2 Dlgap2 +/+ (WT) and Dlgap2 -/- (KO) mice. [file 2040-2392-5-32-S1.docx]

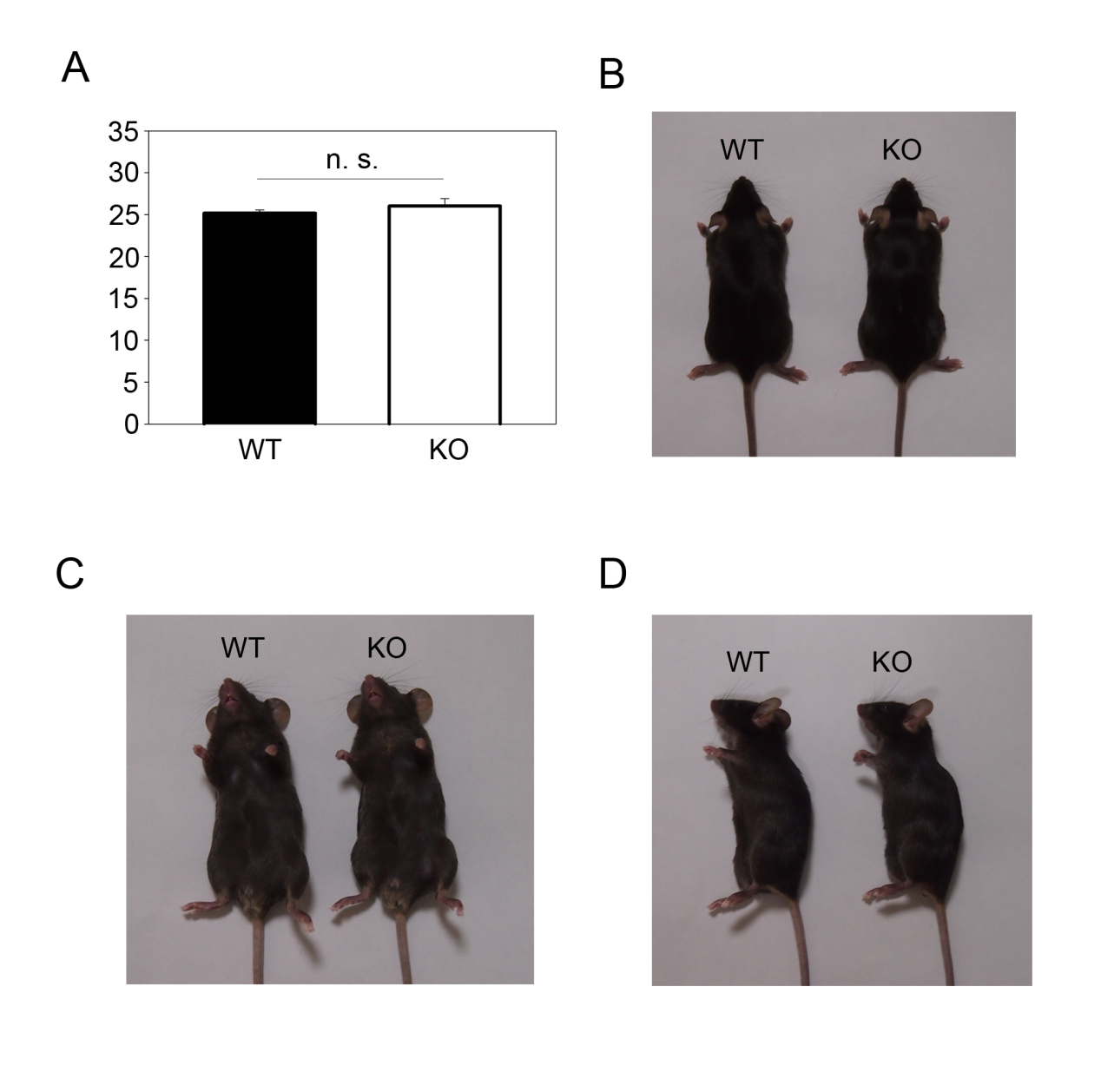


**Additional file 1: Figure S1.** Similar body weight and shape of *Dlgap2^+/+^* (WT) and *Dlgap2^-/-^* (KO) mice. (A) Similar body weight of adult (9 weeks of age) *Dlgap2^+/+^* (WT) and *Dlgap2^-/-^* (KO) mice. Data are presented as mean ± s.e.m., *n* = 10 for each genotype. n. s, not significant, two-tailed t-test. (B-D) Representative pictures of similar body shape and fur conditions of *Dlgap2^+/+^* (WT) and *Dlgap2^-/-^* (KO) mice at 9 weeks of age.


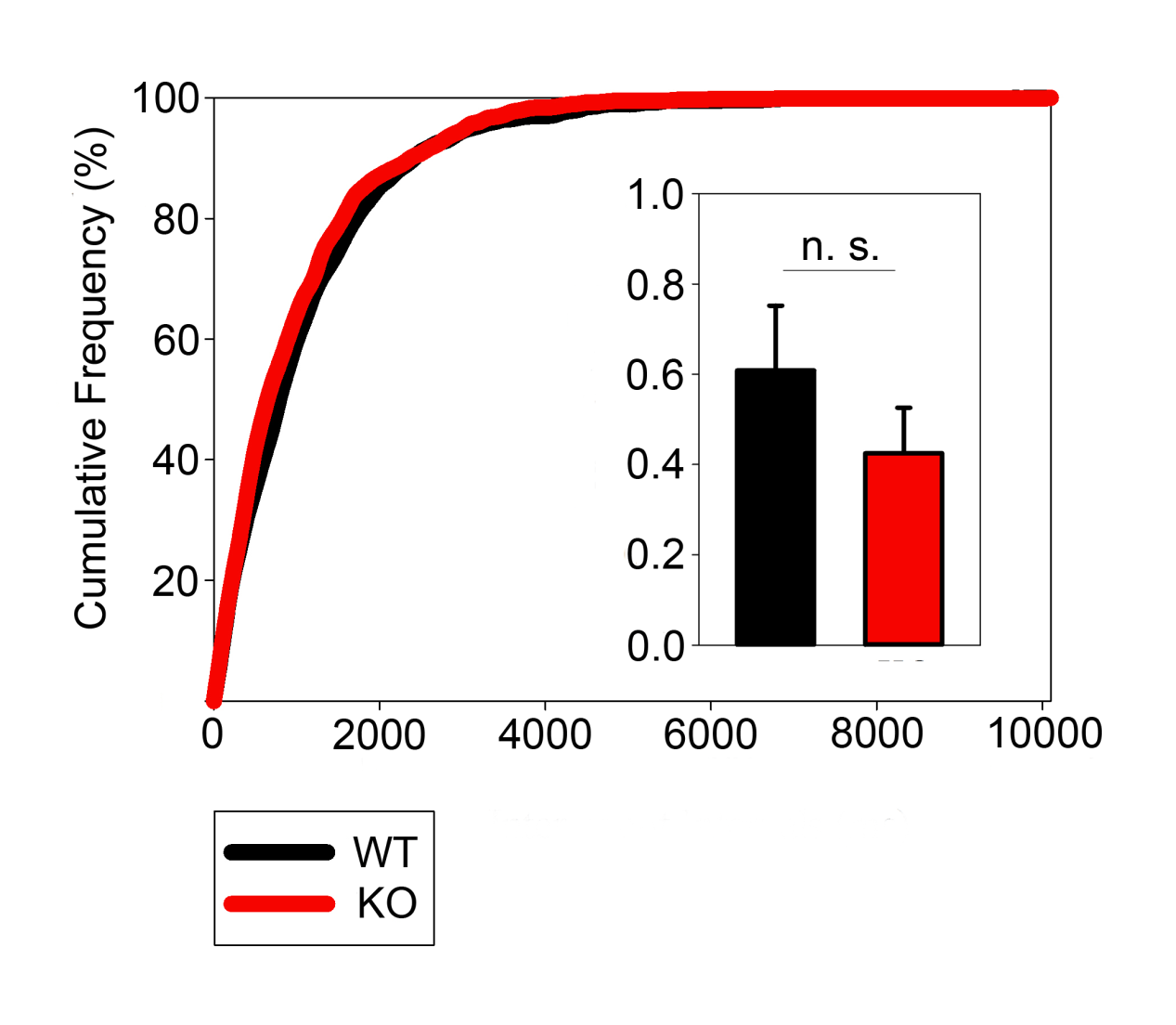


**Additional file 1: Figure S2.** No significant effect on mEPSC frequency in *Dlgap2^-/-^* (KO) mice. Although there is a trend of reduced frequency of mEPSC, it is not statistically significant, *n* = 30~35 neurons from 5 *Dlgap2^+/+^* (WT) and *Dlgap2^-/-^* (KO) mice. n. s, not significant, two-tailed t-test.
